# ACP-DA: Improving the Prediction of Anticancer Peptides Using Data Augmentation

**DOI:** 10.3389/fgene.2021.698477

**Published:** 2021-06-30

**Authors:** Xian-gan Chen, Wen Zhang, Xiaofei Yang, Chenhong Li, Hengling Chen

**Affiliations:** ^1^School of Biomedical Engineering, South-Central University for Nationalities, Wuhan, China; ^2^Hubei Key Laboratory of Medical Information Analysis and Tumor Diagnosis & Treatment, South-Central University for Nationalities, Wuhan, China; ^3^Key Laboratory of Cognitive Science (South-Central University for Nationalities), State Ethnic Affairs Commission, Wuhan, China; ^4^College of Informatics, Huazhong Agricultural University, Wuhan, China; ^5^Hubei Engineering Technology Research Center of Agricultural Big Data, Wuhan, China

**Keywords:** anticancer peptide prediction, data augmentation, feature representation, multilayer perception, machine learning

## Abstract

Anticancer peptides (ACPs) have provided a promising perspective for cancer treatment, and the prediction of ACPs is very important for the discovery of new cancer treatment drugs. It is time consuming and expensive to use experimental methods to identify ACPs, so computational methods for ACP identification are urgently needed. There have been many effective computational methods, especially machine learning-based methods, proposed for such predictions. Most of the current machine learning methods try to find suitable features or design effective feature learning techniques to accurately represent ACPs. However, the performance of these methods can be further improved for cases with insufficient numbers of samples. In this article, we propose an ACP prediction model called ACP-DA (Data Augmentation), which uses data augmentation for insufficient samples to improve the prediction performance. In our method, to better exploit the information of peptide sequences, peptide sequences are represented by integrating binary profile features and AAindex features, and then the samples in the training set are augmented in the feature space. After data augmentation, the samples are used to train the machine learning model, which is used to predict ACPs. The performance of ACP-DA exceeds that of existing methods, and ACP-DA achieves better performance in the prediction of ACPs compared with a method without data augmentation. The proposed method is available at http://github.com/chenxgscuec/ACPDA.

## Introduction

With the increase in population age, cancer has become one of the most threatening diseases for humans (Bray et al., 2018; Zhang et al., 2020). The complexity and heterogeneity of cancer make it difficult to treat. Traditional clinical methods such as surgery, radiotherapy, and chemotherapy can be used to treat cancer, but the side effects of these methods are very obvious and can cause great discomfort for patients (Doja et al., 2020). Although traditional anticancer drugs are effective, their shortcomings, such as gastrointestinal damage (Mitchell, 2006), are also notable and can easily cause multidrug tumor resistance (Holohan et al., 2013; Wijdeven et al., 2016). In view of these problems, it is urgent to find and design novel cancer treatments and anticancer agents to fight cancer. In recent years, due to their high specificity, low production cost, and low toxicity profile, peptides have emerged as alternative anticancer agents (Otvos, 2008).

Anticancer peptides (ACPs), a class of naturally occurring important defense substances, provide a new direction for research involving novel anticancer drugs. ACPs are usually short peptides with a length of 10–50 amino acids. Since ACPs only interact with the anionic cell membrane components of cancer cells, they exhibit extensive cytotoxicity against a variety of cancer cells but not normal cells (Barras and Widmann, 2011; Boohaker et al., 2012). There are currently many peptide-based therapies being evaluated for their efficacy in treating tumors. However, only a few peptides can be used for clinical treatment. Therefore, the discovery of new ACPs is of great significance to the successful clinical application of these peptides. An increasing number of ACPs from protein sequences have been identified and verified by experiments (Tyagi et al., 2013), but it is time consuming and expensive to use experimental methods to identify ACPs. Therefore, computational methods for ACP identification are urgently needed.

There are many computational methods in the field of bioinformatics, that are used to solve different kinds of bioinformatics problems (Zou et al., 2018; Zou, 2019; Deng et al., 2020; Huang et al., 2020). There are many computational methods, especially machine learning-based methods, for the identification of ACPs. Anti-CP was the first computational tool based on a support vector machine (SVM), which used sequence-based features and binary profiles (Tyagi et al., 2013). Hajisharifi et al. (2014) considered Chou’s pseudo amino acid composition (PseAAC) and local alignment kernel for the prediction of ACPs (Amanat et al., 2020; Hasan et al., 2020; Naseer et al., 2020). Chen et al. (2016) developed a method based on the optimization of g-gap dipeptide components. Li and Wang (2016) selected the amino acid composition, average chemical shifts, and reduced amino acid composition to represent ACPs. Wei et al. (2018) developed a feature representation learning model with a two-step feature selection technique to improve the prediction of ACPs. Xu et al. (2018) proposed using 400-dimensional features with g-gap dipeptide features for ACPs. Boopathi et al. (2019) applied a two-step method to obtain optimal feature vectors, which were used as inputs for a SVM. Ge et al. (2019) proposed a generalized chaos game representation (CGR) for ACP identification. Ge et al. (2020) used different features and multiple classifiers and the classifier outputs were used as inputs for a SVM, which was used to identify ACPs. Yu et al. (2020) explored three different deep-learning architectures and found that recurrent neural networks are superior to other architectures. Zhao et al. (2020) used a deep belief network to encode the sequences and chemical features of ACPs and applied random relevance vector machines to identify ACPs. Yi et al., 2019 proposed a deep learning long short-term memory (LSTM) neural network model called ACP-DL to predict novel ACPs. Agrawal et al., 2020 used various features and different machine learning classifiers on two datasets for the prediction of ACPs.

However, the number of ACPs involved in the above methods did not exceed 1000 cases, which is not a large number. The performance of these methods could potentially be further improved if additional ACPs are considered. In this article, we use data augmentation to increase the number of samples in the training set and further improve the performance of ACP prediction methods based on machine learning. Specifically, we propose an ACP prediction model with Data Augmentation, named ACP-DA. In our method, binary profile features (BPFs) and the features that describe the physicochemical properties of amino acids are concatenated to represent peptides, and the samples in the training set are augmented in the feature space. The samples after data augmentation are used to train a machine learning model, which is used for the prediction of ACPs.

**FIGURE 1:**
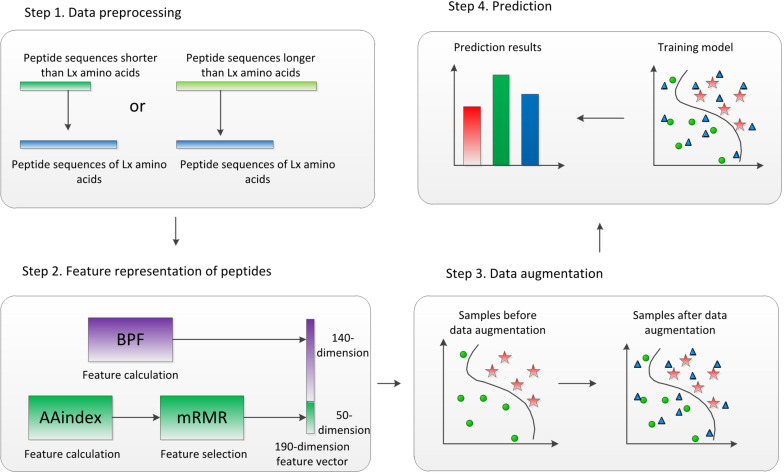
Flowchart of ACP-DA. Binary profile features (BPFs) and AAindex features after feature selection were concatenated to represent peptides, and the samples in the training set were augmented in the feature space. The samples after data augmentation were used to train the multilayer perception (MLP) model, which was used for the prediction of anticancer peptides (ACPs).

The flowchart of ACP-DA is shown in Figure 1. There are four major steps in our method. First, given peptide sequences as the input, each sequence is preprocessed to equal length. Second, the peptide sequences are represented by concatenating BPFs and AAindex features selected based on minimum redundancy maximum relevance (mRMR). Third, data augmentation is performed in the feature space of samples in the training set. Ultimately, the data-augmented samples are used to train a multilayer perception (MLP) model, and the trained MLP model assigns labels to the samples in the testing set. To evaluate the performance of our method, we used five-fold cross-validation to evaluate ACP-DA based on two benchmark datasets: ACP740 and ACP240. We discuss the performance of this method with different parameters and evaluate the effect of data augmentation based on different classifiers. The experimental results show that data augmentation can help improve the prediction of ACPs under the condition of using suitable classifiers, and our method is suitable for ACP prediction.

## Materials and Methods

### Datasets

A good dataset is very important for establishing a reliable ACP prediction model. In recent years, several excellent datasets have been established (Wei et al., 2018; Yi et al., 2019). We selected two benchmark datasets, ACP740 and ACP240, which have more samples (Yi et al., 2019) than others. The similarities between the two datasets were as follow: ACPs verified in the experiment were regarded as positive samples, and anti-microbial peptides (AMPs) without anticancer function were regarded as negative samples. CD-HIT was used to remove the peptide sequences with a similarity of more than 90%. The difference was that ACP740 was from Chen et al.’s and Wei et al.’s studies, while ACP240 was from Yi et al.’s studies. There were 376 positive samples and 364 negative samples in ACP740, and there were 129 positive samples and 111 negative samples in ACP240. There were no overlapping data between ACP740 and ACP240, and both are non-redundant datasets. These two datasets are available at https://github.com/haichengyi/ACP-DL.

### Prediction Framework

To identify potential ACPs, we propose an ACP prediction model called ACP-DA. Figure 1 illustrates the framework of the proposed method. First, we preprocess the peptide sequences to equal length, and the length is selected to be *L_X* amino acids so that the next feature calculation can be performed. Second, the AAindex in the iFeature Python package (Chen et al., 2018) is used to calculate the physicochemical properties of the amino acids in each sequence, and mRMR (Peng et al., 2005) is then used for feature selection. BPFs and AAindex features after feature selection for each peptide sequence are concatenated to represent a peptide. Third, data augmentation is performed in the feature space of samples in the training set for subsequent processing. Finally, the data-augmented samples are used to train the MLP model; the trained MLP model assigns labels to the samples in the testing set. The following sections describe the steps in our framework in detail.

### Preprocessing

**FIGURE 2:**
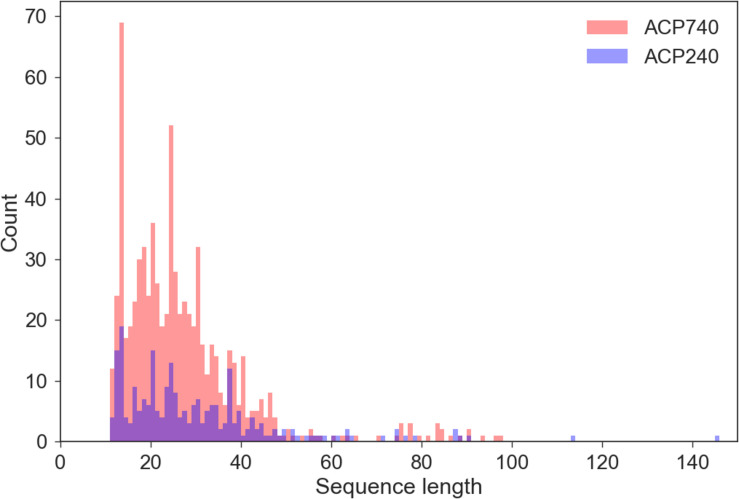
Sequence length statistics for all peptides in the ACP740 and ACP240 dataset.

Since the AAindex in the iFeature Python package can only encode peptides of the same length, we need to preprocess the original peptide sequences to obtain peptides of the same lengths. To obtain the best sequence length, we need to know the length distribution of all samples. We performed statistical analyses of the length of the peptides in the ACP740 and ACP240 datasets. As shown in Figure 2, most of the peptides were less than 60 amino acids in length. To obtain peptides of the same length, we processed each peptide as follows. For sequences less than *L_X* amino acids, each peptide was padded with “*X*” until *L_X* amino acids were reached. For sequences greater than *L_X* amino acids, the extra amino acids after *L_X* were removed, and only the first *L_X* amino acids were reserved. *L_X* can be selected as 40, 50, or 60. We think the best length can be derived from the three numbers.

### Representation of Peptides

The main objective of feature representation is to convert peptides of different lengths into fixed-length feature vectors (Zhang and Liu, 2019). The unprocessed peptide sequence *P* can be represented as:

$$P=p_{1}\,p_{2}\,\ldots \,p_{L}$$

where *p*_*1*_ is the first residue of *P* and *p_L* is the last residue of *P*. *L* is the length of *P*. *p_i* (1≤*i*≤*L*) is an element of the standard amino acid alphabet {A, C, D, E, F, G, H, I, K, L, M, N, P, Q, R, S, T, V, W, Y}. After preprocessing, the peptide sequence can be expressed as:

$$P\;=\;p_{1}p_{2}\ldots p_{L_{X}}$$

Different feature representation methods describe different properties of peptide sequences. If two features have complementary characteristics, combining the two features will help to improve the performance of the predictive model. However, more features don’t necessarily lead to better performance. Too many features may cause redundancy, resulting in performance degradation. So, we tested three feature representation methods and their concatenations: BPFs, AAindex, and K-mer sparse matrix.

#### Binary Profile Features

There are 20 different amino acids in the standard amino acid alphabet. In BPFs, each amino acid is encoded by a feature vector composed of zeroes and ones. The first amino acid type A in the above amino acid alphabet is encoded as f(A)=(1, 0,…, 0), the second amino acid type C is encoded as f(C)=(0, 1,…, 0), and so on. For each given peptide, the N-terminus of *k* amino acids is encoded as the following feature vector:

$$F_{B\,P\,F}=\left[f\,\left(p_{1}\right),f\,\left(p_{2}\right),\ldots ,f\,\left(p_{k}\right)\right]$$

Experiments derived from ACP-DL (Yi et al., 2019) show that the result is best when *k* is 7, which means that only the first 7 amino acids in each peptide sequence are encoded; therefore, the dimension of *F*_*BPF*_ is 20×7=140.

#### AAindex

The physicochemical properties of amino acids represent the characteristics of biochemical reactions and have been widely used in bioinformatics research. The AAindex is a database of amino acid indices representing the physicochemical and biochemical properties of amino acids (Kawashima et al., 2008). We used the AAindex in the iFeature Python package to represent peptides. The AAindex descriptor can only be used to encode peptides of equal length (Tung and Ho, 2008). The preprocessing steps in the previous section changed peptides of different lengths to peptides of equal length for encoding based on the AAindex. If *L_X* in the above section is set to 40, the AAindex descriptor calculated for a peptide of 40 amino acids will result in a 21,240-dimensional feature vector. The dimension of the features is too large, which may cause dimension disaster. mRMR is an excellent dimension reduction technology, and it has good application properties in many scenarios. Therefore, we selected the 50 most informative candidate features by using mRMR for more efficiency.

#### K-mer Sparse Matrix

K-mer of peptides is composed of *K* amino acids. Suppose the length of a peptide sequence is *L*, there will be 7^*K*^ different possible K-mer and an *L*−*K*+1 step appearing in the sequence. One peptide sequence is transformed to a 7^*K*^×(*L*−*K*+1) K-mer sparse matrix *M*, which is a low-rank matrix. Singular value decomposition (SVD) is used to convert this matrix into a 343-dimension feature vector.

A new feature vector is formed to represent peptides by concatenating the above feature representations of each peptide. If BPFs and AAindex are selected, the dimension of the new feature vector is 140 + 50 = 190.

### Data Augmentation

When using machine learning technology to solve scientific problems, insufficient data (Han et al., 2020) or data imbalance (Fu et al., 2020; Gao et al., 2020; Mahmud et al., 2021) issues are common. Collecting more data can certainly solve these problems, but sometimes it may be difficult to obtain more data due to cost restrictions or other reasons. In such cases, data augmentation can potentially be efficient. Data augmentation has mainly historically been used in the field of computer vision (Chaitanya et al., 2021; Wang et al., 2021), and novel samples can be obtained by flipping, rotating, scaling, and cropping the original samples in the methods of data augmentation. In the field of bioinformatics, there will sometimes be data imbalances (Zou et al., 2016; Wan et al., 2017; Meng et al., 2019). Data augmentation can be used to solve data imbalance problems (Chen et al., 2020). Here, we are facing the insufficient sample problem, which can be solved by data augmentation. Four oversampling techniques are used to generate new samples in feature space to improve the performance of the RNA coding potential prediction model (Chen et al., 2020). Noise adding oversampling (NAO) is the best. We also use this technique to generate new samples.

To improve the performance of the ACP prediction model, we augmented the positive and negative samples in the datasets, respectively. Data augmentation is achieved by adding perturbation values to the original samples in the feature space to generate pseudosamples. The features of a peptide include two parts: BPFs and the AAindex. BPFs are binary codes composed of 0 and 1, which are not suitable for adding perturbations. So, we only add perturbations to the AAindex, and the BPFs remain unchanged. A new sample *F*_*new*_ is generated by the following mathematical formula:

$$F_{\mathrm{new}}=F_{i}*V*a+F_{i}$$

where *F_i* is a random sample from the training samples of peptide sequences and *i*=1,…,*N*. *N* is the total number of positive (negative) samples. *V* is a 190-dimensional vector for generating perturbations that corresponds to *F_i*. Because perturbations are not added to BPFs, *V* is composed of two parts: one is a 140-dimensional vector of zeros corresponding to BPFs, and the other is a 50-dimensional random vector with values between 0 and 1 corresponding to AAindex. Thus, perturbations are added to AAindex features, and BPFs are kept unchanged in the pseudosample set *F*_*new*_. *a* is the coefficient of the perturbation and is set to 0.005 for the ACP740 dataset. We repeat the sampling process *N* times to obtain *N* new samples.

### Classifier

The MLP classifier is an artificial neural network composed of an input layer, a hidden layer, and an output layer. The hidden layer can be a single layer or multiple layers, and the layers are fully connected. A back propagation (BP) algorithm is used to train the MLP classifier (Rumelhart et al., 1986). Due to its excellent classification performance, this classifier has been used in many fields of bioinformatics, as noted in Auer et al. (2020). For implementation, we used the scikit-learn Python package; the hidden layer was composed of 6 sublayers, each with 100 neurons. The L2 penalty (regularization term) parameter was 0.01, and the other parameters were set to default values. We employed the MLP classifier to train our predictive model. In this work, we also tested other classifiers, including SVM (Fan et al., 2008), random forest (RF) (Breiman, 2001), MLP, decision tree (DT), and extremely randomized trees (ExtraTrees) (Geurts et al., 2006) classifiers, to build prediction models based on the augmented data in the training set. Among these classifiers, the MLP classifier works best according to the experiments section.

### Performance Evaluation

We used five-fold cross-validation to evaluate the performance of ACP-DA. In the evaluation, five metrics were used in the binary classification tasks. The five metrics were accuracy (ACC), precision (PRE), sensitivity (SN), specificity (SP), and the Matthews correlation coefficient (MCC), which were widely used in bioinformatics (Zhang et al., 2019). These metrics are defined as follows:

$$A\,C\,C=\frac{T\,P+T\,N}{T\,P+T\,N+F\,P+F\,N}$$

$$P\,R\,E=\frac{T\,P}{T\,P+F\,P}$$

$$S\,N=\frac{T\,P}{T\,P+F\,N}$$

$$S\,P=\frac{T\,N}{T\,N+F\,P}$$

$$M\,C\,C=\frac{T\,P*T\,N-F\,P*T\,N}{\sqrt{\left(T\,P+F\,N\right)*\left(T\,P+F\,P\right)*\left(T\,N+F\,P\right)*\left(T\,N+F\,N\right)}}$$

where TP stands for true positives and FN, TN, and FP stand for false negatives, true negatives, and false positives, respectively. MCC is a comprehensive performance evaluation metric.

## Results

In this section, we first discuss the effects of two important parameters on the performance of our method and then compare the performance of the model for different features. We also analyze the effect of data augmentation in the case of using different classifiers. Finally, we compare our method with existing methods.

### Parameter Discussion

Two parameters affect the performance of the model. One is *L_X* in the preprocessing step, which is the length of the peptide sequence after preprocessing. *L_X* can be set to 40, 50, or 60. The other parameter is *N*, which is related to the number of new positive (negative) samples in the data augmentation step. Here, we use the training samples after data augmentation to build the prediction model with 100, 200, or 300% of the original positive (negative) sample number as new samples. Thus, *N* may be set to 100, 200, or 300% of the original positive (negative) sample number.

For the ACP740 and ACP240 datasets, the performance of the prediction models established based on different parameters is shown in Tables 1, 2. MCC is a comprehensive performance evaluation metric, and larger MCC values mean better performance. Therefore, we choose the best parameters *L*_*X*_ 40 and *N* 100% for ACP740 and *L*_*X*_ 40 and *N* 300% for ACP240 according to the maximum MCC value. The N value of ACP240 is larger than that of ACP740, which means that more pseudosamples are needed for ACP240 than ACP740 because ACP240 has fewer samples than ACP740.

**TABLE 1:** Performance of ACP-DA with different parameters based on ACP740 (The best metrics are in bold).

***L*_*X*_**	***N***	**ACC (%)**	**PRE (%)**	**SE (%)**	**SP (%)**	**MCC (%)**
40	100%	81.89	**84.14**	80.59	83.23	**64.71**
40	200%	82.02	83.46	80.89	**83.26**	64.56
40	300%	81.49	82.89	80.88	82.15	63.40
50	100%	80.41	83.35	79.02	81.88	62.59
50	200%	**82.03**	81.51	84.57	79.36	64.68
50	300%	80.27	77.23	**86.98**	73.35	61.17
60	100%	79.19	80.18	79.54	78.85	58.89
60	200%	78.37	77.72	81.67	75.01	57.21
60	300%	79.73	79.14	81.93	77.47	59.61
						

**TABLE 2:** Performance of ACP-DA with different parameters based on ACP240 (The best metrics are in bold).

***L*_*X*_**	***N***	**ACC (%)**	**PRE (%)**	**SE (%)**	**SP (%)**	**MCC (%)**
40	100%	85.42	83.43	92.28	77.59	71.57
40	200%	87.92	87.17	91.48	83.91	76.03
40	300%	**88.33**	**90.11**	88.37	**88.30**	**76.68**
50	100%	85.00	84.71	88.43	81.11	70.10
50	200%	83.75	84.80	86.12	81.10	68.10
50	300%	85.42	86.48	86.86	83.83	71.03
60	100%	86.25	84.35	92.28	79.37	72.97
60	200%	87.08	86.89	90.74	83.04	74.64
60	300%	87.92	85.70	**93.78**	81.11	76.26

### Comparisons With Different Features

Binary profile feature and k-mer sparse matrix have been proved to be effective in ACP-DL (Yi et al., 2019), and AAindex has also been mentioned in physicochemical property based therapeutic peptide predictor (PPTPP) (Zhang and Zou, 2020). BPF and AAindex were introduced in the previous subsection. The k-mer sparse matrix was proposed to represent protein sequences (You et al., 2016), and later used in the representation of peptide sequences (Yi et al., 2019). To obtain more effective features or feature combinations, we use the MLP classifier to build ACP prediction models and test the performance of each model based on three features and their pairwise concatenations without data augmentation.

**FIGURE 3:**
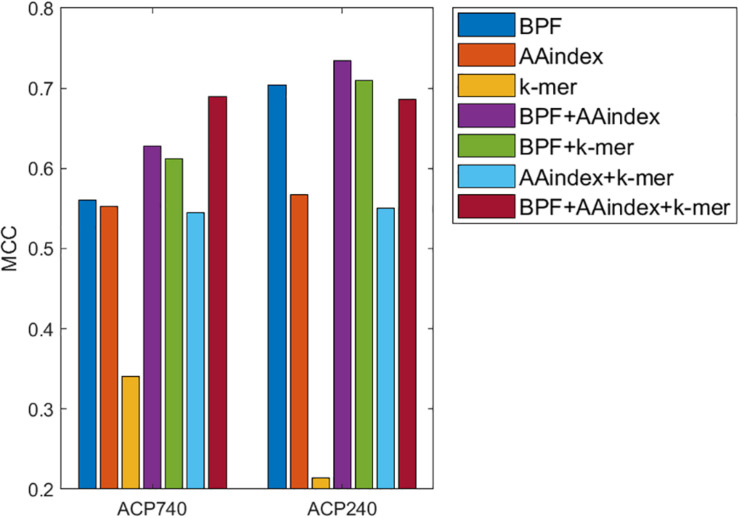
Comparison of prediction models using BPFs, the AAindex, the k-mer sparse matrix (k-mer), and their concatenations based on ACP740 and ACP240.

The three features are BPFs, the AAindex, and the k-mer sparse matrix (k-mer). The concatenations of the three features are BPF + AAindex, BPF + k-mer, AAindex + k-mer and BPF + AAindex + k-mer. The performance of the models for different features and feature concatenations is shown in Figure 3. When the three features are used alone, BPF and AAindex yield the best performance. Among the four feature concatenations, BPF + AAindex yields the best performance for ACP240 and BPF + AAindex + k-mer yields the best performance for ACP740. The performance of BPF + AAindex + k-mer on ACP240 is even worse than that of BPF alone. On the basis of comprehensive consideration of various factors, we chose the concatenation of BPF + AAindex to represent the peptide sequence.

### Classifier Discussion

**FIGURE 4:**
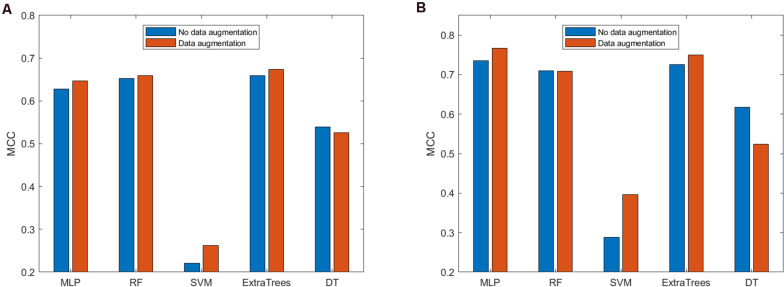
Comparison of the prediction models with and without data augmentation based on **(A)** ACP740 and **(B)** ACP240.

After determining that the concatenation of BPF + AAindex should be used to represent peptides, we need to consider which classifier is the best in our method. We analyzed the performance of the prediction model with data augmentation on several different classifiers. We considered five different classifiers, namely, SVM, RF, MLP, ExtraTrees, and DT classifiers, to build the prediction models. Since MCC is a comprehensive metric, we used it to evaluate the performance of the prediction models. The performance of the models on ACP740 and ACP240 is shown in Figure 4.

As shown in Figure 4A, based on the ACP740 dataset, for the prediction models built using the MLP, RF, SVM, and ExtraTrees classifiers, data augmentation can improve the prediction performance according to the MCC value. However, data augmentation will cause the performance to decrease for the prediction model established based on the DT. As shown in Figure 4B, for the ACP240 dataset, data augmentation can improve the performance of the prediction models established based on the MLP, SVM, and ExtraTrees classifiers and cause the prediction performance of the models based on the RF and DT classifiers to decrease. Therefore, when using the MLP, SVM, and ExtraTrees classifiers, data augmentation is helpful for improving the performance of the ACP prediction model. These results show that the effectiveness of data augmentation is related to the choice of classifier. RF and DT classifiers are not suitable for our prediction model.

As deep learning technology has the advantages of strong learning ability and good portability, it has outstanding performance in various fields in recent years. Combined with the MCC value of the two datasets, we chose the MLP classifier to build the final predictive model.

### Comparison With Existing Methods

**FIGURE 5:**
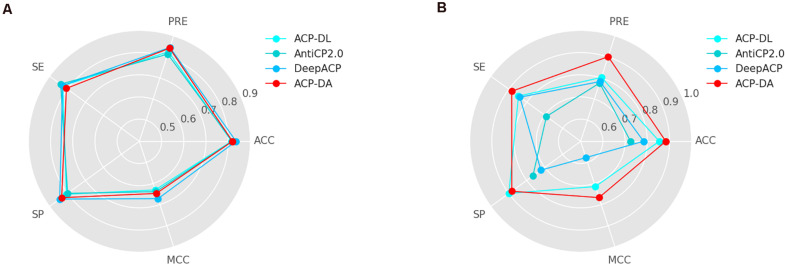
Comparison of ACP-DA with existing methods on **(A)** ACP740 and **(B)** ACP240.

To verify the effectiveness of our proposed method, we compared our method ACP-DA with ACP-DL (Yi et al., 2019), AntiCP 2.0 (Agrawal et al., 2020), and DeepACP (Yu et al., 2020). The results on ACP740 and ACP240 are shown in Figure 5.

Compared with ACP-DL, the advantage of our method lies in the use of data augmentation. In addition, our method used the AAindex feature instead of the k-mer sparse matrix in ACP-DL. Our method with data augmentation outperforms ACP-DL in most metrics, especially on the two metrics of most importance MCC and ACC.

As shown in Figure 5A, the performance of our method on ACP740 was better than that of ACP-DL and AntiCP2.0 and worse than that of DeepACP according to the MCC value and ACC value. Figure 5B shows that our method performed better than other methods on ACP240. The number of samples on ACP240 was less than that on ACP740. Our method performed better on ACP240, which indicated that our method was more suitable for the case of insufficient samples.

## Discussion

As a complex disease, cancer involves complex biological processes. The complex mechanisms of cancer make it difficult to trace the cause. Despite the emergence of various cancer treatment strategies, most of the strategies have been unsatisfactory. Due to its high specificity, high tissue penetration, low production cost and other advantages, treatment based on ACPs has become a potential cancer treatment method. Most ACPs come from protein sequences. The development of high-throughput sequencing technology has brought an increase in the number of available proteins, and it is expected that the number of ACPs will also increase. It is time-consuming and expensive to use experimental methods to discover ACPs from protein sequence data. Therefore, it is urgent to develop computational methods to speed up the identification of ACPs.

In this paper, an ACP prediction method called ACP-DA is proposed. According to the results on the two datasets of ACP740 and ACP240, our model has good overall performance. Compared with existing methods, our method has a better effect in identifying whether the peptide sequence is ACP, and its accuracy may be attributed to the following reasons.

First, how to use effective feature representation methods to characterize peptide sequences is a major challenge in current prediction methods. To find an effective feature or feature combination, we tested 3 feature representation methods and their feature combinations: BPF, AAindex, k-mer, BPF + AAindex, BPF + k-mer, AAindex + k-mer, BPF + AAindex + k-mer. Experiments on the ACP740 and ACP240 datasets show that BPF + AAindex obtains the best performance, so we use BPF + AAindex to represent the peptide sequences.

Second, we used data augmentation to increase the samples in the training set for the insufficient samples. Data augmentation is achieved by generating pseudosamples based on the original samples. The specific method of generating pseudosamples is to add disturbances in the feature space of the original sample. The feature space of the sample is formed by the concatenation of BPF and AAindex. BPF is a code composed of 0 and 1, which is not suitable for adding disturbance, so, we only add disturbance on AAindex to generate pseudosamples. The model is trained with the augmented data to further improve the performance of the prediction model.

Finally, various classifiers show good performance in many classification tasks of bioinformatics. However, it is still unknown whether our data augmentation method can improve the performance of prediction models under various types of classifiers. Therefore, we tested the effect of this method in the case of using five different classifiers. The results show that data augmentation is effective when using MLP, SVM, and ExtraTrees, and data augmentation may not be effective when using RF or DT. Therefore, we choose the MLP with the best overall performance as the final classifier.

The main innovation of this article lies in the use of data augmentation methods. From the experimental results, the method is of great significance. When using MLP, SVM, and ExtraTrees as classifiers, the use of data augmentation can significantly improve the performance of the prediction model. Moreover, a comparative analysis with other methods shows that ACP-DA is better than other methods in most cases.

In short, we provide a new idea for the identification of ACPs, and hope that ACP-DA will play an important role in the development of new anticancer drugs.

## Conclusion

In this work, we proposed a novel ACP prediction model called ACP-DA. To establish an effective prediction model, we concatenated BPFs and the AAindex to represent peptide sequences. We performed data augmentation in the feature space and used the augmented data to train the prediction model. The experimental results show that the proposed method can effectively distinguish ACPs and non-ACPs. Compared with the method without data augmentation, ACP-DA achieves better performance. ACP-DA will be a useful tool for the discovery of novel potential ACPs.

## Data Availability Statement

Publicly available datasets were analyzed in this study. This data can be found here: https://github.com/haichengyi/ACP-DL.

## Author Contributions

X-GC conceived the algorithm, performed the experiments, analyzed the data, and drafted the manuscript. WZ designed the experiments and revised the manuscript. XY, CL, and HC provided suggestions for the study design and the writing of the manuscript. All authors approved the final manuscript.

## Conflict of Interest

The authors declare that the research was conducted in the absence of any commercial or financial relationships that could be construed as a potential conflict of interest.
